# Supplementary material for: Empirical methods for controlling false positives and estimating confidence in ChIP-Seq peaks
Source: BMC Bioinformatics. 2008 Dec 5;9:523. doi: 10.1186/1471-2105-9-523 (PMC2628906; doi:10.1186/1471-2105-9-523)
Supplement: Additional File 1 — A variety of html documents from the USeq web site detailing the available applications, their best usage, output file type descriptions, command line menus, etc. [file 1471-2105-9-523-S1.zip › USeqUserGuides/JavaDoc.zip!/JavaDoc/expr/package-summary.html]

expr


|  |  |  |
| --- | --- | --- |
| **Overview** \| **Package** \| Class \| **Use** \| **Tree** \| **Deprecated** \| **Index** \| **Help** | |  |
| **PREV PACKAGE**   **NEXT PACKAGE** | **FRAMES**    **NO FRAMES**     **All Classes** |  |


---

## Package expr

Classes and tools related to expression array analysis.

**See:**
  
          **Description**

| **Class Summary** | |
| --- | --- |
| **CorrelationMaps** |  |
| **CorrelationWindow** |  |
| **ExpressedGene** |  |
| **ExpressionScores2CoordinatesViaGFF** |  |
| **FileCrossFilter** |  |
| **FileMatchJoiner** |  |
| **MiRNA** |  |
| **MiRNATarget** |  |
| **NormalizeAgilentData** | Takes two replicas, parses out those probes that are not 1's, median centers, then averages ProbeName ProbeName LogRatio gIsPosAndSignif rIsPosAndSignif LogRatio gIsPosAndSignif rIsPosAndSignif A\_51\_P100021 A\_51\_P100021 -0.087965 1 1 0.344989 0 1 A\_51\_P100034 A\_51\_P100034 0.038444 1 1 0.034538 1 1 A\_51\_P100052 A\_51\_P100052 -0.146955 1 0 0 0 0 |
| **ParseAgilentData** | Parses Agilent data based on a list of column names. |
| **ParseManipulateColumnDataForGeneSifter** |  |
| **ParseMiRNATable** |  |
| **ParseWormMart** |  |
| **StripZeros** |  |

## Package expr Description

Classes and tools related to expression array analysis.

---


|  |  |  |
| --- | --- | --- |
| **Overview** \| **Package** \| Class \| **Use** \| **Tree** \| **Deprecated** \| **Index** \| **Help** | |  |
| **PREV PACKAGE**   **NEXT PACKAGE** | **FRAMES**    **NO FRAMES**     **All Classes** |  |


---
